# Prostate-specific antigen screening at low thresholds of men with pathogenic *BRCA1/2* variants

**DOI:** 10.1038/s41391-025-00938-z

**Published:** 2025-01-21

**Authors:** Hein V. Stroomberg, Klaus Brasso, Anna A. Blak, Anna Byrjalsen, Thomas van Overeem Hansen, Andreas Røder

**Affiliations:** 1 Copenhagen Prostate Cancer Center, Department of Urology, Copenhagen University Hospital – Rigshospitalet, Copenhagen, Denmark; 2 Biotech Research & Innovation Center (BRIC), University of Copenhagen, Copenhagen, Denmark; 3 Department of Clinical Medicine, University of Copenhagen, Copenhagen, Denmark; 4 Department of Clinical Genetics, Copenhagen University Hospital – Rigshospitalet, Copenhagen, Denmark

**Keywords:** Cancer genetics, Diagnostic markers, Cancer screening, Diagnostic markers

## Abstract

**Background:**

Men with pathogenic *BRCA1/2* variants are at higher risk of prostate cancer We included men with likely pathogenic/pathogenic (LP/P) variants in *BRCA1/2* in a prostate-specific antigen (PSA) screening program after cascade germline testing since 2014. PSA was tested yearly and an age-specific low PSA threshold for biopsy was used, to determine if a low PSA threshold for biopsy is justified for men with pathogenic *BRCA1/2* variants.

**Methods:**

From 2014 to 2023 a total of 340 men were included in the program. We report demographics, clinical characteristics, and treatment outcomes at 7 years.

**Results:**

The cumulative incidence of a primary biopsy was 37% (95CI: 31‒43) after 7 years. Incidence of prostate cancer diagnosis was 11% (95CI: 7.1‒15). Men referred were 7.8 (95CI: 5.3‒11, *p* < 0.001) times more likely to be diagnosed with prostate cancer than the general Danish male population. The cumulative incidence of biochemical failure (PSA > = 0.2 ng/ml) 4 years after RP was 22% (95CI: 2.3‒41). The main limitation is that not all men underwent a pre-biopsy MRI.

**Conclusion:**

We found a high incidence of prostate cancer in men with LP/P *BRCA1/2* variants, but this may be explained by the low PSA threshold for scheduling biopsies. More studies are needed to compare this patient population to men with other germline features. The high risk of recurrence after curative therapy is worrisome and requires further evaluation as to whether this is a biological phenomenon.

## Introduction

Breast Cancer Genes 1 and 2 (*BRCA1/2*) are involved in DNA homologous recombination repair and are typically included in germline gene panels for testing genetic predisposition to cancer [1]. People with germline likely pathogenic/pathogenic (LP/P) *BRCA1/2* variants are at an increased risk of various cancers, including breast, ovarian, pancreatic, and prostate cancers [2, 3]. Continuous innovations in sequencing have led to increased genomic profiling, and carriers of LP/P *BRCA1/2* variants have been the focus of tailored screening programs [4–6]. Emerging evidence of germline predisposition to cancer has led to cascade testing of family members, who may be referred for closer follow-up [2, 3, 7, 8]. Prostate cancer is considered genetically driven owing to evidence of high heritability, and LP/P *BRCA1/2* variants have been of specific interest [9–11].

Men can be referred for genetic counseling based on familial LP/P *BRCA1/2* variants, fulfilled criteria for genetic screening of familial breast and/or ovarian cancer, high-grade prostate cancer before the age of 50, or a family history of prostate cancer [12, 13]. This has led to an increasing number of men in Denmark being tested for germline predisposition, including *BRCA1/2*. Denmark had no guidelines for early detection of cancer in men with LP/P *BRCA1/2* variants until consensus guidelines for early detection of prostate cancer were implemented in 2023 [12]. In 2014, we decided to offer all men with LP/P *BRCA1/2* variants a prospective screening program with annual prostate-specific antigen (PSA) testing, and we reported the outcomes with 8 years of follow-up. The objective of this study was to investigate whether age-specific low PSA thresholds for the detection of prostate cancer should be used in men with pathogenic *BRCA1/2* variants.

## Methods

We included all men carrying an LP/P *BRCA1/2* variant in the Capital Region Denmark (~1.8 million people) surveillance. Testing for LP/P *BRCA1/2* variants was performed at the DANAK-accredited Center for Genomic Medicine, Rigshospitalet. *BRCA1/2* variants were classified according to *BRCA1/2* gene-specific American College of Medical Genetics and Genomics and the Association for Molecular Pathology (ACMG/AMP) guidelines version 1.1.0 [14]. The PM2_supporting criteria were based on data from gnomAD (V.2.1.1). All men carrying an LP/P *BRCA1/2* variant were counseled on their increased risk of prostate cancer at the Department of Clinical Genetics and were subsequently referred to the Department of Urology, Rigshospitalet, to enter the screening program. At referral, the men underwent an initial physical examination, including transrectal ultrasound and recording of previous medical history. A family history of cancer was recorded up to second-degree relatives self-reported by the subject. When the number of relatives was recorded as multiple, they were recorded as two family members. Subsequently, the men were followed up with annual PSA testing. Routine diagnostic work-up was performed based on age and PSA levels, with biopsy considered at PSA levels of 0.75, 1.0, or 1.5 ng/ml for men aged <50, 50–59, and >59 years, respectively [15]. All men with PSA levels above the threshold or suspicious findings on routine digital rectal examination/ultrasonography were offered a transrectal biopsy at the first visit. Magnetic resonance imaging (MRI) after biopsy indication based on increased PSA levels was gradually introduced in the work-up, and in recent years, all men have undergone MRI before biopsy. However, biopsies were performed even with normal MRI if otherwise indicated by either clinical judgment or PSA level, as the value of MRI in *BRCA1/2* carriers remains unknown. Gleason grading followed the ISUP guidelines at the time of histopathological assessment. Men treated with radical prostatectomy (RP) or radiation had PSA-level follow-up, using standard criteria for progression, e.g., PSA ≥ 0.2 ng/ml for RP. Clinical data were extracted from the electronic health records. The study was approved by the Institutional Review Board (R-23066301; P-2023-15060). Oral informed consent was provided by the participants at the time of referral. Data reporting followed the STROBE reporting guidelines.

### Statistics

All men were followed until 31-01-2024 or death. The cumulative incidences of biopsy, prostate cancer diagnosis, and other cancer diagnoses were estimated from the time of referral until the event, end of follow-up, or death, whichever came first by the Aalen-Johansen estimator for competing risks. The time from RP to biochemical failure was analyzed using the Kaplan-Meier estimate from the time of operation. Time to death was estimated using the Kaplan Meier estimate from the time of referral. Median follow-up was defined as the time to censoring. A multivariable cause-specific Cox proportional hazards model was used to identify baseline variables associated with the risk of prostate cancer diagnosis, with death as a competing risk. The multivariable model included the type of LP/P variant, age at referral, PSA level, PSA density, family history of prostate cancer, and number of family members with cancer. The underlying timeframe was from the time of referral to prostate cancer diagnosis, death, or the end of follow-up. Proportionality was assessed using the Schoenfeld residuals. The nonlinear univariable predicted risk of PSA and PSA density was assessed using a cause-specific Cox Proportional hazard model, with nonlinearity achieved by restricted cubic splines with two internal knots. Age- and calendar-year corrected standardized incidence ratios (SIR) were calculated for prostate cancer and non-prostate cancer malignancies using population-based numbers from 2014-2021 of NORDCAN 9.1 as reference, and for death with the Danish male population from 2014-2023 as reference [16, 17]. Incidence rates per person-year among the cohort were calculated based on the number of events within each year with age groups on January 1st of each year until prostate cancer diagnosis, death, or the end of follow-up. The Byar approximation was used to calculate the 95% confidence intervals (95CI) and p-values of the SIR. All reported p-values are two-sided, a *P*-value below 0.05 was deemed statistically significant, and analyses were performed using R version 4.2.2 on R studio version 2022.07.1

## Results

### Baseline characteristics

A total of 340 men with a median age of 52 years (IQR: 44–62) were included. Of the men, 175 had an LP/P *BRCA2* variant. One man had both pathogenic *BRCA1* and *BRCA2* variants. Fifty-four different *BRCA1* and 70 different *BRCA2* variants were identified; all but five were described in the ClinVar database (Supplementary Table 1) [18]. Of the men, 8.5% had a history of malignancies, 16 men had suspicious findings at the first clinical assessment, family history of prostate cancer and breast cancer was recorded in 28% and 82% of the patients, respectively, the median PSA level at referral was 0.9 ng/ml (IQR: 0.6‒1.9), and prostate volume 24 ml (IQR: 19‒33; Table 1). The median follow-up was 5.1 years (IQR: 2.9‒7.5).

**Table 1:** Clinical characteristics at referral for men carrying a likely pathogenic/pathogenic variant in *BRCA1/2*.

Variable		*BRCA1 (*N = *165)*	*BRCA2* (*N* = *176)*)	All (*N* = 340)^a^
Age (Years), Median (IQR)		53 (45–62)	51 (44–61)	52 (44–62)
Previous cancer diagnosis, *N* (%)	Yes	6 (3.6%)	24 (14%)	29 (8.5%)
	No	159 (96%)	152 (86%)	311 (92%)
Family history of prostate cancer, *N* (%)^b^	Yes	42 (25%)	55 (31%)	97 (29%)
	No	123 (75%)	121 (69%)	243 (71%)
Family history of breast cancer, *N* (%)^b^	Yes	119 (79%)	139 (85%)	257 (82%)
	No	31 (21%)	24 (15%)	55 (18%)
	Unknown	15	13	28
Family history of ovarian cancer, *N* (%)^b^	Yes	53 (35%)	37 (23%)	90 (29%)
	No	97 (65%)	126 (77%)	222 (71%)
	Unknown	15	13	28
Family history of other cancer, *N* (%)^b^	Yes	40 (27%)	53 (33%)	92 (19%)
	No	110 (73%)	110 (67%)	220 (71%)
	Unknown	15	13	28
Number of family members with cancer, median (IQR)		2 (1–3)	2 (2–3)	2 (1–3)
	Missing	10	8	18
Prostate volume (ml), median (IQR)		23 (19–31)	25 (20–34)	24 (19–33)
	Missing	16	5	21
PSA at referral (ng/ml), median (IQR)		0.9 (0.6–1.5)	0.9 (0.6–1.5)	0.9 (0.6–1.5)
PSA density (ng/ml/ml), median (IQR)		0.04 (0.03–0.06)	0.04 (0.03–0.06)	0.04 (0.03–0.06)
	Missing	16	5	21
Clinical assessment at initial visit	Suspicious	10 (6.1%)	6 (3.4%)	16 (4.7%)
	Benign	154 (64%)	167 (97%)	321 (95%)
	Missing	1	2	3
Sterilized prior to referral, *N* (%)	Yes	24 (15%)	29 (16%)	53 (16%)
	No	141 (85%)	147 (84%)	287 (84%)
Biopsy, *N*	Yes	44	65	109
	No	121	111	231
Number of biopsies, *N* (%)	1	33 (75%)	54 (83%)	87 (80%)
	>1	11 (25%)	11 (17%)	22 (20%)
Number of PSA measurements before biopsy, median (IQR)		2 (1–2)	2 (1–5)	2 (1–4)
Number of PSA measurements without biopsy, median (IQR)		4 (3–7)	4 (2–6)	4 (2–7)
MRI prior to diagnosis, *N* (%)	Yes	33 (20%)	46 (26%)	79 (23%)
	No	132 (80%)	130 (74%)	261 (77%)
PIRADS score prior to diagnosis, *N* (%)	1	13 (39%)	11 (24%)	24 (30%)
	2	10 (30%)	20 (43%)	30 (38%)
	3	1 (3.0%)	3 (6.5%)	4 (5%)
	4	7 (21%)	11 (24%)	18 (23%)
	5	2 (6.1%)	1 (2.2%)	3 (4%)

*BRCA1/2* Breast cancer genes 1 and 2, *N* Number, *IQR* Interquartile range, *PSA* Prostate-specific antigen, *MRI* Magnetic resonance imaging, *PIRADS* Prostate imaging-reporting and data systems.

^a^ One patient carried a *BRCA1 and* *BRCA2* variant.

^b^ Patient reported family history.

### Cancer detection

**Fig. 1:**
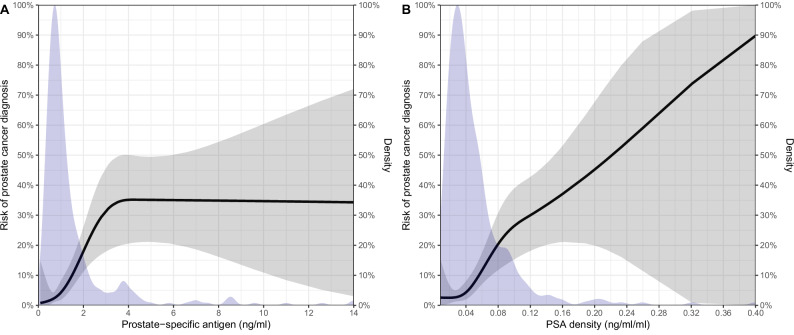
The association of prostate-specific antigen at referral with risk of prostate cancer. Predicted risk of prostate cancer based on prostate-specific antigen (PSA) (**A**)/PSA density (**B**) at referral and density of PSA (**A**)/PSA density (**B**) measurements at referral.

The median number of PSA measurements before the initial biopsy was 2 (IQR: 1-4), with the PSA level increasing from referral, to diagnosis depending on age (Supplementary Table 2). Seven years after referral, the cumulative incidence of biopsy, prostate cancer diagnosis, and other cancer diagnoses was 37% (95CI: 31‒43), 11% (95CI: 7.1‒15), and 10% (95CI: 5.8‒15), respectively (Supplementary Fig. 1). When comparing LP/P *BRCA1* and *BRCA2* variants, only the incidence of biopsy was statistically different (*P* = 0.03, Gray’s test, Supplementary Fig. 2). For every 2-fold increase in PSA, the risk of prostate cancer diagnosis increased 1.6 times (95CI: 1.1‒2.4, *P* = 0.01), and per 0.1 ng/ml/ml increase in PSA density, the risk of prostate cancer diagnosis increased 1.9 times (95CI: 0.97‒3.7, *P* = 0.06), and none of the other included variables were associated with increased prostate cancer risk (Table 2). Nonlinear modeling of PSA and PSA density showed that the risk of prostate cancer plateaued at a PSA level of 4 ng/ml and a linear association with PSA density (Fig. 1). The SIR of prostate cancer was 7.8 (95CI: 5.3‒11, *P* < 0.001), 5.5 (95CI: 2.9‒9.4, *P* < 0.001), and 11 (95CI: 6.6‒17, *P* < 0.001) for men, men with an LP/P *BRCA1* variant, and men with an LP/P *BRCA2* variant, respectively. The age-specific SIR for prostate cancer diagnosis was the highest in younger men (Supplementary Table 3). The SIR for all cancers other than prostate was 1.4 (95CI: 0.91‒2.1, *P* = 0.12).

**Table 2:** Association of baseline variables with prostate cancer diagnosis, and the competing risk of death.

Variable		Prostate cancer diagnosis		Death	
		*Hazard ratio (95CI)*	*P-value*	*Hazard ratio (95CI)*	*P-value*
Gene	*BRCA1*	Reference		Reference	
	*BRCA2*	1.35 (0.62‒2.9)	0.44	4.9 (0.59‒41)	0.14
Age (per year)		1.01 (0.97‒1.05)	0.60	1.25 (1.11‒1.40)	<0.001
PSA (per doubling ng/ml)		1.63 (1.11‒2.4)	0.01	0.60 (0.25‒1.45)	0.26
PSA density (per 0.1 ng/ml/ml increase)		1.89 (0.97‒3.7)	0.06	2.2 (0.24‒20)	0.48
Family history of Prostate cancer	No	Reference		Reference	
	Yes	1.75 (0.81‒3.8)	0.16	1.88 (0.29‒12)	0.51
Number of family members with cancer (per family member)		1.13 (0.90‒1.44)	0.30	1.41 (0.88‒2.3)	0.16

*BRCA1/2* Breast cancer genes 1 and 2, *PSA* Prostate-specific antigen, *95CI* 95% confidence interval.

### Diagnostic characteristics

The 32 men diagnosed with prostate cancer during follow-up had a median age of 62 years (IQR: 54‒66), a median PSA of 2.8 ng/ml (IQR: 2.0‒6.0), and 67% had an ISUP grade of group 1 at diagnosis. The majority (63%) had low-risk disease according to D’Amico, all had clinically localized disease, and 72% were treated with radical prostatectomy (Table 3) [19]. Of the men with an LP/P *BRCA1* variant, 85% had low-risk disease at diagnosis, and 47% of the men with an LP/P *BRCA2* variant had low-risk disease at diagnosis (Supplementary Table 4). Men treated with RP predominantly had ISUP 1 on biopsy (73%), 38% of men were upgraded on RP specimens, and 5 men with pT2 had positive surgical margins.

**Table 3:** Biopsy and radical prostatectomy characteristics of the men diagnosed with prostate cancer.

Men diagnosed with prostate cancer (*N* = 32)		
*Variable*		
Age, years, median (IQR)		62 (54‒66)
PSA at time of biopsy, ng/ml, median (IQR)		2.8 (2.0‒6.0)
Clinical stage, *N* (%)	T1c	22 (69)
	T2a	6 (19)
	T2b+c	3 (9.4)
	T3	1 (3.1)
Number of biopsies before diagnosis, *N* (%)	1	26 (81)
	>1	6 (19)
Diagnostic Gleason grade, *N* (%)	ISUP 1	22 (69)
	ISUP 2	5 (16)
	ISUP 3	2 (6.3)
	ISUP 4	1 (3.1)
	ISUP 5	2 (6.2)
Systematic biopsy Gleason grade, *N* (%)	Non-malignant	3 (9.4)
	ISUP 1	21 (66)
	ISUP 2	4 (13)
	ISUP 3	1 (3.1)
	ISUP 4	1 (3.1)
	ISUP 5	2 (6.3)
Number of cores, *N*, median (IQR)		10 (10‒10)
Number of positive cores, *N*, median (IQR)		1 (1‒3)
Total tumor length, mm, median (IQR)		1.5 (0.9‒4.1)
Total biopsy length, mm, median (IQR)		136 (112–159)
PIRADS prior, *N* (%)	No MRI	17 (53)
	1‒2	3 (9.4)
	4	10 (31)
	5	2 (6.3)
Targeted biopsy Gleason grade, *N* (%)	Non-malignant	2 (18)
	ISUP 1	6 (55)
	ISUP 2	2 (18)
	ISUP 3	1 (9.1)
	ISUP 4‒5	0
	Not taken	2
Number of cores, *N*, median (IQR)		3 (3‒4)
Number of positive cores, *N*, median (IQR)		2 (1‒2)
Total tumor length, mm, median (IQR)		3 (0.8‒7)
Total biopsy length, mm, median (IQR)		39 (32‒47)
D’Amico risk category, *N* (%)	Low	20 (63)
	Intermediate	8 (25)
	High	4 (13)
Primary treatment, *N* (%)	Acitve surveillance	5 (16)
	Radical prostatectomy	24 (75)
	Radiation therapy	1 (3.1)
	Hormonal therapy	2 (6.3)

Men treated by radical prostatectomy (*N* = 26)		
*Variable*		
Biopsy Gleason grade, *N* (%)	ISUP 1	18 (69)
	ISUP 2	6 (23)
	ISUP 3	1 (3.8)
	ISUP 4	1 (3.8)
Prostatectomy Gleason grade, *N* (%)	ISUP 1	9 (35)
	ISUP 2	12 (46)
	ISUP 3	5 (19)
Gleason grade change, biopsy to Prostatectomy, *N* (%)	Downgrading	1 (3.8)
	Same	15 (58)
	Upgrading	10 (38)
Prostate volume, g, median (IQR)		34.5 (32.0‒40.8)
Tumor volume, %, median (IQR)		5 (1.6‒10)
Multifocal tumor, *N* (%)	Yes	24 (92)
	No	2 (7.7)
pT, *N* (%)	pT2	23
	pT3a/b	3
pN, *N* (%)	N0	2 (7.7)
	NX	24 (92)
Margin status, *N* (%)	Positive	5 (19)
	Negative	21 (81)
MSKCC post-operative predicted 5-year biochemical failure-free rate, %, median (IQR)		95 (89‒98)

*N* Number, *IQR* Interquartile range, *PSA* Prostate-specific antigen, *MRI* Magnetic resonance imaging, *PIRADS* Prostate imaging-reporting and data systems, *ISUP* International Society of Urological Pathology, *MSKCC* Memorial Sloan Kettering Cancer Center.

### Oncological outcomes

The cumulative incidence of biochemical failure 4 years after RP was 22% (95CI: 2.3‒41). At the last follow-up, the man who opted for radiation therapy had unmeasurable PSA, and no distant metastases occurred in any of the men. The cumulative incidence of death was 8.5% (95CI: 3.1‒14) 10 years after referral for all men, and none of the men died of prostate cancer. The SIR for death in the entire cohort was 0.69 (95CI: 0.36‒1.2, *P* = 0.15).

## Discussion

Generally, men are referred for prostate diagnostic workup by general practitioners based on elevated PSA levels [20]. Cascade-tested family members comprise a unique group of typically healthy individuals who are referred to cancer screening programs; however, evidence for such a strategy is scarce. Prostate cancer screening in men with LP/P *BRCA2* variants is recommended, predominantly based on a single study with a PSA threshold for biopsy of 3 ng/ml [21]. The recommendation for the early detection of men with LP/P *BRCA1/2* variants is further based on observations that oncological outcomes are poorer than expected in these men, even in localized disease [22, 23]. Here, we report the initial results of a systematic screening with a low PSA threshold indication for biopsy in men with LP/P *BRCA1/2* variants.

The *BRCA1/2* positive men referred were typically younger than those referred by general practitioners [24]. When comparing our results to those of recent population-based screening studies in men of similar age, we found a consistently higher biopsy rate (37% compared to 0.8% in the PROBASE trial, 0.93% in the OPT study, and 5.8% in the GÖTEBORG-2 trial), lower detection of cancer on biopsy (29% vs. 40% in the PROBASE trial, 62% in the OPT study, and 40% in the GÖTEBORG-2 trial), and a greater proportion of ISUP 1 cancer (69% compared to 13% in the PROBASE trial, 32% in the OPT study, and 51% in the GÖTEBORG-2 trial) [25–27]. These major differences can be explained by the lower PSA threshold for biopsy in our study and the use of pre-biopsy MRI in the OPT study to reduce the number of ISUP 1 diagnoses. Furthermore, compared to the age-matched Danish population, prostate cancer was diagnosed eight times more often than expected in men with LP/P *BRCA1/2* variants, similar to the SIR of five observed in men with initial non-malignant biopsies [28]. This similar SIR implies a surveillance bias, but an increased risk of poorly differentiated prostate cancer and prostate cancer-specific death in *BRCA2*, 999del5 (c.771_775del) carriers indicates an underlying biological explanation [29]. In line with previous research, we demonstrated that both men with LP/P *BRCA1* and *BRCA2* variants are at a high risk of prostate cancer diagnosis, with the highest risk among men carrying LP/P variants in *BRCA2* [21, 30]. The higher SIR compared to the previous study (2.4 (1.4‒3.8) vs. 5.5 (2.9‒9.4), and 4.5 (3.0‒6.6) vs. 11 (6.6‒17) for *BRCA1* and *BRCA2* positive men, respectively) likely reflects our systematic diagnostic work-up with low PSA values. Notably, the frequency of ISUP 2 or higher at diagnosis was 66% in men with prostate cancer in the largely non-screened setting, which is higher than the observed 31%. Furthermore, the IMPACT study, a screened population of men with LP/P *BRCA1/2* variants with a PSA threshold of 3 ng/ml for biopsy showed similar cancer incidence rates of 14 and 19 per 1000 person-years compared to our 14 and 23 per 1000 person-years for men with LP/P variants in *BRCA1* and *BRCA2*, respectively [21]. We detected prostate cancer ISUP 2 or higher diagnosis in 8% and 47% of men with LP/P *BRCA1* and *BRCA2* variants, respectively, compared to the IMPACT study showing ISUP 2 or higher diagnosis in 45% and 63% of men with *BRCA1* and *BRCA2* variants, respectively. In conclusion, the low PSA threshold is potentially problematic due to the high number of biopsies performed and a high proportion of diagnosed low-grade cancers, which challenges our approach for detecting prostate but not other cancers in these men. As the clinical course of prostate cancer in men with LP/P *BRCA1/2* variants may be worse, the detection of low-grade cancer in men with LP/P *BRCA1/2* variants needs to be weighed against the treatment outcomes [22, 23].

Based on the diagnostic characteristics of our cohort, many men were candidates for active surveillance, according to European guidelines [31]. However, the optimal treatment strategy for these patients remains unknown [32, 33]. Because of previous reports of potentially poor oncological outcomes and the aim to decrease disease mortality, we advocated for treatment in this research setting [22, 23]. We observed a substantial risk of upgrading from biopsy to RP specimen assessment in our early treatment setting, in line with previous research [22, 23]. Moreover, we observed a relatively high biochemical failure rate in men with favorable post-RP 5-year risk predictions, indicating that carriers of LP/P *BRCA1/2* variants with localized prostate cancer could be at a higher risk of progressive disease. However, this finding warrants confirmation, as previous research on oncological outcomes of treatment for men with localized prostate cancer and an LP/P *BRCA1/2* variant is conflicting [22, 23, 34, 35]. The positive surgical margin rate was high and required internal reflection but potentially explained the biochemical failure rate. The post-RP 5-year risk predictions, which included margin status, indicated a 5-year risk of biochemical failure of 5%. Therefore, the margin status is unlikely to be the only explanation for the relatively high biochemical failure rate observed. However, even systematic biopsies in men with small prostates seem to undergrade the tumor. Whether MRI can change this requires further exploration because of uncertainties regarding MRI in men with small prostates and low PSA levels. We did not observe prostate cancer-related mortality, likely due to the relatively short follow-up. The remaining question is whether men with LP/P *BRCA1/2* variants harbor a biologically aggressive form of prostate cancer that requires earlier detection and treatment than the general population. Prostate cancer is typically defined as a genomic heterogenic disease, and it is speculated that these monogenic cohorts separate themselves from other prostate cancer patients. Certainly, data point to the fact that disease is more prevalent in younger ages than expected, and we can speculate that these cancers can suddenly progress into lethal diseases due to their inherent problem with DNA damage repair during cellular replication [36]. It is evident that larger international collaborations are needed and that diagnostic tests and programs should be tested in randomized clinical trials for this specific patient population.

Our study was limited by constantly changing clinical guidelines during the inclusion period. The use of MRI as a screening tool is promising and can potentially identify men with prostate cancer without increased PSA levels. Data on MRI as a triage test are lacking; however, if early detection is important for men with LP/P *BRCA1/2* variants, many MRIs will likely be normal, as ISUP 1 was primarily found on systematic biopsies. A study has suggested that MRI is favorable in the diagnostic pathway for men with LP/P *BRCA1/2* variants; however, that study used MRI as a complimentary tool for whom to biopsy, not as a selection tool [37]. It could be considered a limitation that not all men over the age-specific threshold underwent biopsy, however, the median PSA at initial diagnosis is below the normal median PSA at diagnosis in Denmark [24]. Furthermore, higher PSA at diagnosis compared to the time of biopsy reflects the differences in prostate cancer detection rate based on PSA level, and previous systematic screening studies also reported PSA levels >4 ng/ml in 12.5% of patients at the time of first screening [38]. Moreover, we want to emphasize that the final decision to perform biopsy was always a shared decision; therefore, some chose not to undergo biopsy. Another limitation is the relatively small number of men included, which hampers our conclusions regarding biochemical failure following RP and survival. However, since our results are in line with previous results, we believe that our conclusions are unaltered. The number of men-prevented sub-analyses separated for LP or P variants and for common LP/P variants in specific regions of *BRCA1/2* genes. Specific pathogenic variants are likely worse than others, which could refine future follow-up strategies. Furthermore, future studies should elucidate whether variants of unknown significance are pathogenic, increasing the population size that could benefit from screening. The strengths of our study include the completeness of data, stringent follow-up of the included men, prospective research setting, and early implementation of referral for men with LP/P *BRCA1/2* variants.

## Conclusion

Our study of men with LP/P *BRCA1/2* variants, followed by a yearly PSA screening program, indicated a higher than expected number of men with prostate cancer in the early stages at a young age but also observed poor oncological outcomes. Whether men with LP/P *BRCA1/2* variants harbor more aggressive prostate cancer remains uncertain. However, with the expected increase in cascade-tested men, trials investigating systematic screening programs and risk stratification are urgently needed to ensure proper management in this unique population.

## Supplementary information

Supplementary material

## Data Availability

The data generated and/or analyzed during the current study are available for research purposes upon reasonable request from the corresponding author.
