# Supplementary material for: Prostate-specific antigen screening at low thresholds of men with pathogenic BRCA1/2 variants
Source: Prostate Cancer Prostatic Dis. 2025 Jan 21;28(4):894–901. doi: 10.1038/s41391-025-00938-z (PMC12643936; doi:10.1038/s41391-025-00938-z)
Supplement: Supplementary file 1 — Supplementary material [file 41391_2025_938_MOESM1_ESM.docx]

**Supplementary Table 1.** Detailed description of the frequency of Likely Pathogenic/Pathogenic *BRCA1/2* variants in the cohort.

| ***BRCA1*** | | | | |
| --- | --- | --- | --- | --- |
| *Variant* | *N* | *Amino acid* | *Assessment* | *Class* |
| c.2475del | 19 | p.(Asp825Glufs*21) | PVS1; PM5_PTC_Strong | 5 |
| c.3319G>T | 14 | p.(Glu1107*) | PVS1; PM2_supporting; PM5_PTC_Strong | 5 |
| c.5266dup | 11 | p.(Gln1756Profs*74) | PVS1; PM5_PTC_Strong | 5 |
| c.3400G>T | 9 | p.(Glu1134*) | PVS1; PM5_PTC_Strong | 5 |
| c.(80+1_81-1)_(4986+1_4987-1)del | 8 | p.(Cys27*) | PVS1; PM2_supporting; PM5_PTC_Strong | 5 |
| c.427G>T | 7 | p.(Glu143*) | PP3; PS3; PP1_Strong; PM3_Supporting | 5 |
| c.5096G>A | 7 | p.(Arg1699Gln) | PVS1; PM2_Supporting; PP1 | 5 |
| c.68_69del | 6 | p.(Glu23Valfs*17) | PVS1; PM5_PTC_Strong | 5 |
| c.130T>A | 5 | p.(Cys44Ser) | PP3; PS3; PP1_Strong; PM2_Supporting | 5 |
| c.181T>G | 5 | p.(Cys61Gly) | PP3; PS3; PP1_Strong; Expert panel | 5 |
| c.3048_3052dup | 4 | p.(Asn1018Metfs*8) | PVS1; PM5_PTC_Strong | 5 |
| c.1687C>T | 3 | p.(Gln563*) | PVS1; PM5_PTC_Strong | 5 |
| c.3607C>T | 3 | p.(Arg1203*) | PVS1; PM5_PTC_Strong | 5 |
| c.3710del | 3 | p.(Ile1237Asnfs*27) | PVS1; PM2_supporting; PM5_PTC_Strong | 5 |
| c.3718C>T | 3 | p.(Gln1240*) | PVS1; PM2_supporting; PM5_PTC_Strong | 5 |
| c.4964C>T | 3 | p.(Ser1655Phe) | PP3; PS3; PP1_Strong; PM2_Supporting | 5 |
| c.5089T>C | 3 | p.(Cys1697Arg) | PP3; PS3; PP1_Moderate; PM2_Supporting; PP4_Very_strong | 5 |
| c.5213G>A | 3 | p.(Gly1738Glu) | PP3; PS3; PP1_Strong; PM2_Supporting | 5 |
| c.5503C>T | 3 | p.(Arg1835*) | PVS1; PM5_PTC_Strong | 5 |
| c.1556del | 2 | p.(Lys519Argfs*13) | PVS1; PM2_supporting; PM5_PTC_Strong | 5 |
| c.1823_1826del | 2 | p.(Lys608Ilefs*3) | PVS1; PM5_PTC_Strong | 5 |
| c.220C>T | 2 | p.(Gln74*) | PVS1; PM2_supporting; PM5_PTC_Strong | 5 |
| c.3485del | 2 | p.(Asp1162Valfs*48) | PVS1; PM5_PTC_Strong | 5 |
| c.3700_3704del | 2 | p.(Val1234Glnfs*8) | PVS1; PM2_supporting; PM5_PTC_Strong | 5 |
| c.3753T>A | 2 | p.(Cys1251*) | PVS1; PM2_supporting; PM5_PTC_Strong | 5 |
| c.4222C>T | 2 | p.(Gln1408*) | PVS1; PM2_supporting; PM5_PTC_Strong | 5 |
| c.5143A>C | 2 | p.(Ser1715Arg) | PP3; PS3; PP1_Strong; PM2_Supporting | 5 |
| c.5153-1G>C | 2 | p.(?) | PS3; PM2_Supporting; PVS1_Strong (RNA); PP1 | 5 |
| c.(80+1_81-1)_(134+1_135-1)del | 2 | p.(Cys27*) | PVS1; PM2_Supporting; PP1 | 5 |
| c.843_846del | 2 | p.(Ser282Tyrfs*15) | PVS1; PM2_supporting; PM5_PTC_Strong | 5 |
| c.115T>G | 1 | p.(Cys39Gly) | PS3; PM2_Supporting; PP1; PP3; PS4 | 5 |
| c.132C>T | 1 | p.(Cys44=) | PVS1 (RNA); PS3; PM2_Supporting | 5 |
| c.2110_2111del | 1 | p.(Asn704Cysfs*7) | PVS1; PM2_supporting; PM5_PTC_Strong | 5 |
| c.2275C>T | 1 | p.(Gln759*) | PVS1; PM5_PTC_Strong | 5 |
| c.2393del | 1 | p.(Pro798Glnfs*5) | PVS1; PM2_supporting; PM5_PTC_Strong | 5 |
| c.2477_2492delinsTG | 1 | p.(Thr826Metfs*6) | PVS1; PM2_supporting; PM5_PTC_Strong | 5 |
| c.2685_2686del | 1 | p.(Pro897Lysfs*5) | PVS1; PM5_PTC_Strong | 5 |
| c.2694del | 1 | p.(Val899Serfs*101) | PVS1; PM2_supporting; PM5_PTC_Strong | 5 |
| c.3188_3189delinsG | 1 | p.(Ser1063*) | PVS1; PM2_supporting; PM5_PTC_Strong | 5 |
| c.3377del | 1 | p.(Pro1126Hisfs*3) | PVS1; PM2_supporting; PM5_PTC_Strong | 5 |
| c.3640G>T | 1 | p.(Glu1214*) | PVS1; PM2_supporting; PM5_PTC_Strong | 5 |
| c.3756_3759del | 1 | p.(Ser1253Argfs*10) | PVS1; PM5_PTC_Strong | 5 |
| c.3844G>T | 1 | p.(Glu1282*) | PVS1; PM2_supporting; PM5_PTC_Strong | 5 |
| c.3904G>T | 1 | p.(Glu1302*) | PVS1; PM2_supporting; PM5_PTC_Strong | 5 |
| c.3907_3908delinsGGA | 1 | p.(Leu1303Glyfs*27) | PVS1; PM2_supporting; PM5_PTC_Strong | 5 |
| c.4035del | 1 | p.(Glu1346Lysfs*20) | PVS1; PM5_PTC_Strong | 5 |
| c.(4096+1_4097-1)_(4185+1_4186-1)del | 1 | p.(Glu1366Alafa*8) | PVS1; PM2_Supporting; PP1 | 5 |
| c.4321_4324del ⱡ | 1 | p.(Asp1441Cysfs*14) | PVS1; PM5_PTC_Strong | 5 |
| c.4327C>T | 1 | p.(Arg1443*) | PVS1; PM5_PTC_Strong | 5 |
| c.463C>T | 1 | p.(Gln155*) | PVS1; PM2_supporting; PM5_PTC_Strong | 5 |
| c.4675+1G>A | 1 | p.(?) | PVS1 (RNA); PM2_Supporting; PP1 | 5 |
| c.5123C>A | 1 | p.(Ala1708Glu) | PS3; PP3; PP1; Expert panel | 5 |
| c.(5152-1_5153+1)_(5193+1_5194-1)del | 1 | p.(Trp1718Serfs*2) | PVS1; PM2_Supporting; PP1 | 5 |
| c.5341del | 1 | p.(Glu1781Asnfs*12) | PVS1; PM2_supporting; PM5_PTC_Strong | 5 |
| ***BRCA2*** | | | | |
| *Variant* | *N* | *Amino acid* | *Assessment* | *Class* |
| c.6486_6489del | 19 | p.(Lys2162Asnfs*5) | PVS1; PM5_PTC_Strong | 5 |
| c.6373del | 14 | p.(Thr2125Profs*12) | PVS1; PM2_supporting; PM5_PTC_Strong | 5 |
| c.1310_1313del | 8 | p.(Lys437Ilefs*22) | PVS1; PM5_PTC_Strong | 5 |
| c.7069_7070del | 7 | p.(Leu2357Valfs*2) | PVS1; PM5_PTC_Strong | 5 |
| c.3530_3533del | 6 | p.(Asp1177Alafs*19) | PVS1; PM2_supporting; PM5_PTC_Strong | 5 |
| c.7878G>C | 6 | p.(Trp2626Cys) | PS3; PP3; PM1; Expert panel | 5 |
| c.145G>T | 5 | p.(Glu49*) | PVS1; PM5_PTC_Strong | 5 |
| c.2808_2811del | 5 | p.(Ala938Profs*21) | PVS1; PM5_PTC_Strong | 5 |
| c.3847_3848del | 5 | p.(Val1283Lysfs*2) | PVS1; PM2_supporting; PM5_PTC_Strong | 5 |
| c.5073dup | 5 | p.(Trp1692Metfs*3) | PVS1; PM5_PTC_Strong | 5 |
| c.37G>T | 4 | p.(Glu13*) | PVS1; PM2_supporting; PM5_PTC_Strong | 5 |
| c.516G>A | 4 | p.(Lys172=) | PP1; PVS1_Strong; PM2_Supporting | 4 |
| c.5946del | 4 | p.(Ser1982Argfs*22) | PM3_Strong; PVS1; PM5_PTC_Strong | 5 |
| c.771_775del | 4 | p.(Asn257Lysfs*17) | PVS1; PM5_PTC_Strong | 5 |
| c.2830A>T | 3 | p.(Lys944*) | PVS1; PM5_PTC_Strong | 5 |
| c.316+5G>A | 3 | p.(?) | PVS1 (RNA); PM2_Supporting; P4_Very_strong | 5 |
| c.5164_5165del | 3 | p.(Ser1722Tyrfs*4) | PVS1; PM5_PTC_Strong | 5 |
| c.8953+1G>T | 3 | p.(?) | PVS1 (RNA); PM2_Supporting; PP4_Strong | 5 |
| c.9253dup | 3 | p.(Thr3085Asnfs*26) | PVS1; PM5_PTC_Strong | 5 |
| c.2099T>A | 2 | p.(Leu700*) | PVS1; PM2_supporting; PM5_PTC_Strong | 5 |
| c.2231C>G | 2 | p.(Ser744*) | PVS1; PM2_supporting; PM5_PTC_Strong | 5 |
| c.2376C>G | 2 | p.(Tyr792*) | PVS1; PM2_supporting; PM5_PTC_Strong | 5 |
| c.3751dup | 2 | p.(Thr1251Asnfs*14) | PVS1; PM5_PTC_Strong | 5 |
| c.469_470del | 2 | p.(Lys157Valfs*25) | PVS1; PM2_supporting; PM5_PTC_Strong | 5 |
| c.5219del | 2 | p.(Leu1740*) | PVS1; PM2_supporting; PM5_PTC_Strong | 5 |
| c.6082_6086del | 2 | p.(Glu2028Lysfs*19) | PVS1; PM5_PTC_Strong | 5 |
| c.6275_6276del | 2 | p.(Leu2092Profs*7) | PVS1; PM5_PTC_Strong | 5 |
| c.7006dup | 2 | p.(Arg2336Profs*4) | PVS1; PM2_supporting; PM5_PTC_Strong | 5 |
| c.7913_7917del | 2 | p.(Phe2638*) | PVS1; PM2_supporting; PM5_PTC_Strong | 5 |
| c.8754+3G>C | 2 | p.(?) | PVS1 (RNA); PM2_Supporting | 5 |
| c.9154C>T | 2 | p.(Arg3052Trp) | PS3; PP3; PP1; Expert panel | 5 |
| c.7617+1G>A | 2 | p.(?) | PVS1 (RNA); PM2_Supporting; PP1 | 5 |
| c.1813del | 1 | p.(Ile605Tyrfs*9) | PVS1; PM5_PTC_Strong | 5 |
| c.1929del | 1 | p.(Arg645Glufs*15) | PVS1; PM2_supporting; PM5_PTC_Strong | 5 |
| c.2129C>A ⱡ | 1 | p.(Ser710*) | PVS1; PM2_supporting; PM5_PTC_Strong | 5 |
| c.2450del | 1 | p.(Lys817Argfs*8) | PVS1; PM2_supporting; PM5_PTC_Strong | 5 |
| c.2748T>A | 1 | p.(Cys916*) | PVS1; PM2_supporting; PM5_PTC_Strong | 5 |
| c.2886dup | 1 | p.(Ile963Tyrfs*19) | PVS1; PM2_supporting; PM5_PTC_Strong | 5 |
| c.3265C>T | 1 | p.(Gln1089*) | PVS1; PM2_supporting; PM5_PTC_Strong | 5 |
| c.3599_3600del | 1 | p.(Cys1200*) | PVS1; PM5_PTC_Strong | 5 |
| c.4095T>A | 1 | p.(Cys1365*) | PVS1; PM2_supporting; PM5_PTC_Strong | 5 |
| c.4780del | 1 | p.(Met1594Cysfs*23) | PVS1; PM2_supporting; PM5_PTC_Strong | 5 |
| c.5180dup | 1 | p.(Asn1727Lysfs*2) | PVS1; PM2_supporting; PM5_PTC_Strong | 5 |
| c.5238dup | 1 | p.(Asn1747*) | PVS1; PM2_supporting; PM5_PTC_Strong | 5 |
| c.5352del | 1 | p.(Asn1784Lysfs*7) | PVS1; PM2_supporting; PM5_PTC_Strong | 5 |
| c.5600_5601del ⱡ | 1 | p.(Thr1867Argfs*5) | PVS1; PM2_supporting; PM5_PTC_Strong | 5 |
| c.5682C>G | 1 | p.(Tyr1894*) | PVS1; PM5_PTC_Strong | 5 |
| c.5722_5723del | 1 | p.(Leu1908Argfs*2) | PVS1; PM5_PTC_Strong | 5 |
| c.5754_5755del | 1 | p.(His1918Glnfs*5) | PVS1; PM5_PTC_Strong | 5 |
| c.5857G>T | 1 | p.(Glu1953*) | PVS1; PM5_PTC_Strong | 5 |
| c.6154_6155del | 1 | p.(Ser2052Ilefs*7) | PVS1; PM2_supporting; PM5_PTC_Strong | 5 |
| c.6388_6392del | 1 | p.(Phe2130Ilefs*4) | PVS1; PM5_PTC_Strong | 5 |
| c.6443_6444del | 1 | p.(Ser2148Tyrfs*2) | PVS1; PM2_supporting; PM5_PTC_Strong | 5 |
| c.6408_6414del | 1 | p.(Asn2137Lysfs*29) | PVS1; PM2_supporting; PM5_PTC_Strong | 5 |
| c.(6841+1_6842-1)_(7805+1_7806-1)del | 1 | p.(Glu2282Leufs*46) | PVS1; PM2_Supporting, PP1 | 5 |
| c.7008-1G>A | 1 | p.(?) | PVS1 (RNA); PM2_Supporting; PP1 | 5 |
| c.7165A>T ⱡ | 1 | p.(Arg2389*) | PVS1; PM2_supporting; PM5_PTC_Strong | 5 |
| c.7480C>T | 1 | p.(Arg2494*) | PVS1; PM5_PTC_Strong | 5 |
| c.755_758del | 1 | p.(Asp252Valfs*24) | PVS1; PM5_PTC_Strong | 5 |
| c.7988A>T | 1 | p.(Glu2663Val) | PM2_Supporting; PP1, PP3; PS3; Expert panel | 5 |
| c.8165C>G | 1 | p.(Thr2722Arg) | PM2_Supporting; PP1; PS3; Expert panel | 5 |
| c.8474del | 1 | p.(Ala2825Aspfs*38) | PVS1; PM2_supporting; PM5_PTC_Strong | 5 |
| c.8488_8489del | 1 | p.(Trp2830Aspfs*14) | PVS1; PM2_supporting; PM5_PTC_Strong | 5 |
| c.8575del | 1 | p.(Gln2859Lysfs*4) | PVS1; PM5_PTC_Strong | 5 |
| c.8730del ⱡ | 1 | p.(Lys2791Asnfs*30) | PVS1; PM2_Supporting; Expert panel | 5 |
| c.9106C>T | 1 | p.(Gln3036*) | PVS1; PM2_supporting; PM5_PTC_Strong | 5 |
| c.9196C>T | 1 | p.(Gln3066*) | PVS1; PM2_supporting; PM5_PTC_Strong | 5 |
| c.9352_9353del | 1 | p.(Met3118Valfs*31) | PVS1; PM2_supporting; PM5_PTC_Strong | 5 |
| c.9382C>T | 2 | p.(Arg3128*) | PVS1; PM5_PTC_Strong | 5 |
| c.9401del | 1 | p.(Gly3134Alafs*29) | PVS1; PM2_supporting; PM5_PTC_Strong | 5 |
| ⱡ Denotes a novel variant | | | | |

**Supplementary Table 2.** Prostate-specific antigen (PSA) levels, presented as median with interquartile range, at the initial visit to the urological department, at the time of biopsy, and at the time of diagnosis, stratified for age. The age groups are defined as the age at the different time points..

|  | **Age groups** | | |
| --- | --- | --- | --- |
|  | ***<50*** | ***50-59*** | ***≥60 years*** |
| PSA at referral, ng/ml | 0.8 (0.6 ‒ 1.2) | 0.9 (0.6 – 1.3) | 1.6 (0.6 – 1.5) |
| n | 155 | 90 | 95 |
| PSA at initial biopsy, ng/ml | 1.6 (1.3 ‒ 1.8) | 1.8 (1.4 – 3.1) | 3.9 (2.3 – 6.0) |
| n | 32 | 31 | 46 |
| PSA at diagnosis, ng/ml | 1.8 (1.4 ‒ 1.8) | 2.6 (1.7 – 4.1) | 4.5 (2.9 – 6.9) |
| n | 5 | 9 | 18 |

**Supplementary Table 3.** Calendar year and age-matched standardized prostate cancer incidence ratios (SIR) separated based on age groups. The prostate cancer SIR is depicted for the entire cohort and separately based on *BRCA1/2* gene. Any cancer other than prostate and mortality SIR are age-corrected but shown only for the entire cohort.

| **SIR prostate cancer entire cohort** | | | | | |
| --- | --- | --- | --- | --- | --- |
| *Age groups* | *Person years* | *Events* | *Expected events* | *SIR*  *(95% Confidence interval)* | *p-value* |
| 30‒45 | 280 | 3 | 0.003 | 893 (179‒2,609) | <0.001 |
| 45‒50 years | 332 | 2 | 0.05 | 43 (4.8‒154) | 0.005 |
| 50‒54 years | 288 | 4 | 0.17 | 23 (6.3‒60) | <0.001 |
| 55‒59 years | 259 | 5 | 0.44 | 11 (3.7‒26) | <0.001 |
| 60‒64 years | 185 | 9 | 0.65 | 14 (6.3‒26) | <0.001 |
| 65‒70 years | 208 | 4 | 1.3 | 3.2 (0.85‒8.1) | 0.08 |
| 70+ years | 213 | 5 | 1.5 | 3.3 (1.1‒7.6) | 0.04 |
| Overall | 1,765 | 32 | 4.1 | 7.8 (5.3‒11) | <0.001 |
| **SIR prostate cancer men with LP/P *BRCA1* variant** | | | | | |
| *Age groups* | *Person years* | *Events* | *Expected events* | *SIR*  *(95% Confidence interval)* | *p-value* |
| 30‒45 | 141 | 1 | 0.002 | 591 (7.7‒3,288) | 0.02 |
| 45‒50 years | 148 | 1 | 0.02 | 48 (0.63‒267) | 0.07 |
| 50‒54 years | 131 | 2 | 0.08 | 26 (2.9‒93) | 0.01 |
| 55‒59 years | 139 | 2 | 0.24 | 8.4 (0.95‒30) | 0.05 |
| 60‒64 years | 101 | 6 | 0.35 | 17 (6.2‒37) | <0.001 |
| 65‒70 years | 115 | 1 | 0.70 | 1.4 (0.02‒8.0) | 0.99 |
| 70+ years | 135 | 0 | 0.97 |  |  |
| Overall | 910 | 13 | 2.4 | 5.5 (2.9‒9.4) | <0.001 |
| **SIR prostate cancer men with LP/P *BRCA2* variants** | | | | | |
| *Age groups* | *Person years* | *Events* | *Expected events* | *SIR*  *(95% Confidence interval)* | *p-value* |
| 30‒45 | 139 | 2 | 0.002 | 1,199 (135‒4,329) | <0.001 |
| 45‒50 years | 179 | 1 | 0.03 | 40 (0.52‒220) | 0.07 |
| 50‒54 years | 152 | 2 | 0.09 | 22 (2.5‒80) | 0.01 |
| 55‒59 years | 120 | 3 | 0.20 | 15 (3.0‒43) | 0.004 |
| 60‒64 years | 84 | 3 | 0.30 | 10 (2.0‒30) | 0.009 |
| 65‒70 years | 93 | 3 | 0.56 | 5.3 (1.1‒16) | 0.04 |
| 70+ years | 78 | 5 | 0.56 | 8.9 (2.9‒21) | <0.001 |
| Overall | 845 | 19 | 1.7 | 11 (6.6‒17) | <0.001 |
| **SIR any other than prostate entire cohort** | | | | | |
| *Age groups* | *Person years* | *Events* | *Expected events* | *SIR*  *(95% Confidence interval)* | *p-value* |
| Overall | 1840 | 24 | 17 | 1.4 (0.91‒2.1) | 0.12 |
| **Standardized mortality ratio entire cohort** | | | | | |
| *Age groups* | *Person years* | *Events* | *Expected events* | *SIR*  *(95% Confidence interval)* | *p-value* |
| Overall | 1877 | 12 | 17 | 0.69 (0.36‒1.2) | 0.15 |

**Supplementary Table 4.** Diagnostic characteristics stratified by *BRCA1/2* gene.

| **Variable** | | ***BRCA1* (n=13)** | ***BRCA2* (n=19)** |
| --- | --- | --- | --- |
| Age, years, median (IQR) | | 61 (53‒63) | 62 (55‒70) |
| PSA, ng/ml, median (IQR) | | 2.6 (1.8‒3.9) | 3.9 (2.0‒6.3) |
| Clinical stage, n (%) | T1c | 8 (62) | 14 (74) |
|  | T2a | 4 (31) | 2 (11) |
|  | T2b+c | 1 (8) | 2 (11) |
|  | T3 | 0 | 1 (5) |
| Diagnostic Gleason grade, n (%) | ISUP 1 | 12 (92) | 10 (53) |
|  | ISUP 2 | 1 (8) | 4 (21) |
|  | ISUP 3 | 0 | 2 (11) |
|  | ISUP 4 | 0 | 1 (5) |
|  | ISUP 5 | 0 | 2 (11) |
| PIRADS prior | No MRI | 8 (62) | 9 (47) |
|  | 1-2 | 1 (8) | 2 (1) |
|  | 4 | 3 (23) | 7 (37) |
|  | 5 | 1 (8) | 1 (5) |
| D’Amico risk category, n (%) | Low | 11 (85) | 9 (47) |
|  | Intermediate | 1 (8) | 7 (37) |
|  | High | 1 (8) | 3 (16) |
| Abbreviations: PSA = Prostate-specific antigen; PIRADS = Prostate imaging-reporting and data system | | | |

**Supplementary Figure 1.** Cumulative incidence from time of referral of biopsy (A), prostate cancer diagnosis (B), other cancer diagnosis (C), death (E), and cumulative incidence of biochemical failure after radical prostatectomy (D).





**Supplementary Figure 2.** Cumulative incidence from time of referral of biopsy (A), prostate cancer diagnosis (B), other cancer diagnosis (C), and death (D) stratified by *BRCA1/2* gene.
